# Cognitive Enhancement Therapy Improves Frontolimbic Regulation of Emotion in Alcohol and/or Cannabis Misusing Schizophrenia: A Preliminary Study

**DOI:** 10.3389/fpsyt.2015.00186

**Published:** 2016-01-12

**Authors:** Jessica A. Wojtalik, Susan S. Hogarty, Jack R. Cornelius, Mary L. Phillips, Matcheri S. Keshavan, Christina E. Newhill, Shaun M. Eack

**Affiliations:** ^1^School of Social Work, University of Pittsburgh, Pittsburgh, PA, USA; ^2^Department of Psychiatry, School of Medicine, University of Pittsburgh, Pittsburgh, PA, USA; ^3^Department of Psychiatry, Harvard Medical School, Boston, MA, USA

**Keywords:** schizophrenia, alcohol misuse, cannabis misuse, emotion regulation, brain, cognitive enhancement therapy

## Abstract

Individuals with schizophrenia who misuse substances are burdened with impairments in emotion regulation. Cognitive enhancement therapy (CET) may address these problems by enhancing prefrontal brain function. A small sample of outpatients with schizophrenia and alcohol and/or cannabis substance use problems participating in an 18-month randomized trial of CET (*n* = 10) or usual care (*n* = 4) completed posttreatment functional neuroimaging using an emotion regulation task. General linear models explored CET effects on brain activity in emotional neurocircuitry. Individuals treated with CET had significantly greater activation in broad regions of the prefrontal cortex, limbic, and striatal systems implicated in emotion regulation compared to usual care. Differential activation favoring CET in prefrontal regions and the insula mediated behavioral improvements in emotional processing. Our data lend preliminary support of CET effects on neuroplasticity in frontolimbic and striatal circuitries, which mediate emotion regulation in people with schizophrenia and comorbid substance misuse problems.

## Introduction

Substance misuse among individuals with schizophrenia is substantially higher compared to the general population (1, 2), which has been linked to problems in emotion dysregulation (3–5), defined as the inability to tolerate and appropriately manage emotions, particularly negative affects. Individuals with schizophrenia who misuse substances tend to function worse in the community (6), experience lower quality of life (7), treatment non-adherence (8, 9), and have higher rates of relapse often leading to emergency room contact and/or hospitalization (10). Indeed, the experience of overall greater severity of the illness in this population often results in two to three times more hospitalizations than patients with schizophrenia who do not abuse substances (11). Misuse of substances has been proposed to exacerbate symptoms (12), such that positive symptoms tend to be particularly more severe in people with schizophrenia and comorbid substance misuse diagnoses (13). Cannabis and alcohol have been indicated to be the two most commonly misused substances among individuals with schizophrenia (2). While literature on the relationship between substance misuse and cognitive functioning in schizophrenia have been mixed and difficult to interpret (14), generally it can be inferred that individuals with schizophrenia who misuse alcohol or cannabis experience cognitive deficits (15, 16). In a recent randomized-controlled trial, we found that cognitive enhancement therapy [CET (17)] resulted in significant improvements in emotion regulation abilities among people with schizophrenia and alcohol and/or cannabis use problems, as well as significant reductions in alcohol use (18). CET is a psychosocial cognitive remediation intervention (19) that integrates 60 h of computer-based training targeted at improving attention, memory, and problem solving with 45 structured social-cognitive group sessions designed to improve abilities, such as perspective taking, social context appraisal, and emotion regulation abilities. Examination of the neurobiological underpinnings of improved emotion regulation associated with cognitive remediation can yield important information about the plasticity of neural mechanisms that can support addiction and psychiatric recovery in this population.

The affect regulation model has been one of the most empirically supported conceptualizations of the nature of high rates of substance misuse among individuals with schizophrenia (3, 5). This model proposes that individuals with schizophrenia who are high in trait negative affect are more likely to misuse substances as a way to cope and regulate the intensity of negative emotional states (3, 4). In support of this model, numerous studies have found that people with schizophrenia report that they misuse substances to relieve or buffer dysregulated negative emotions (20, 21). The brain circuitry for supporting successful emotion regulation involves regions in the prefrontal cortex, limbic system, and the striatum (22–24), which are also regions thought to be impacted by the pharmacological effects of addiction (25) and the symptoms of schizophrenia (26). For example, dysfunctional communication between the nucleus accumbens, frontal cortex, and hippocampus observed in non-substance abusing schizophrenia patients is similar to the substance abuse-related neurobiological changes observed in primary additive disorders (26).

Based on the aforementioned findings, we posit that participation in cognitive remediation interventions may alter functioning of the frontal, limbic, and striatal neurocircuitry to support improvement in emotion regulation in individuals with schizophrenia and substance misuse comorbidity. Meta-analytic evidence is supportive of neuroplasticity or “brain changing” effects of cognitive remediation interventions, where individuals with schizophrenia have demonstrated increased brain function in prefrontal, limbic, and striatal regions following treatment (27). Additionally, protection against gray matter loss in limbic regions has been observed in early course schizophrenia outpatients treated with CET (28). Both increased neural activation and gray matter in frontolimbic and striatal regions were associated with improved cognitive and socioemotional outcomes (27, 28). Although substance misuse is often an exclusion criterion in cognitive remediation trials (29), such findings suggest that these treatments, such as CET, may have the ability to strengthen neurobiological functions that govern emotional circuitry in individuals with schizophrenia and substance misuse comorbidity (29). However, no study has examined the neurobiological effects of cognitive remediation in people with schizophrenia who misuse substances.

Identifying biomarkers of therapeutic mechanisms in the treatment of individuals with schizophrenia is imperative for the continued understanding of the pathophysiology of the illness and the impact of substance misuse. More importantly, understanding the neurobiological effects of cognitive remediation could reinforce the utility of such interventions (19) to intervene with the diversity of challenges people with schizophrenia face, including substance misuse. Therefore, this preliminary study sought to explore the posttreatment neurobiological impact of CET on frontolimbic and striatal brain functioning during the effortful regulation of emotion in a small sample of individuals with schizophrenia who misuse alcohol and/or cannabis, the two most commonly abused substances in this population (2). The degree to which posttreatment brain functioning during emotion regulation was related to longitudinal behavioral improvements in emotion processing was also investigated.

## Materials and Methods

### Participants

A total of 14 individuals diagnosed with schizophrenia (*n* = 10) or schizoaffective disorder (*n* = 4) and alcohol and/or cannabis misuse problems were included in an 18-month randomized feasibility study (NCT01292577) of CET compared to treatment as usual [TAU (18)]. All participants provided written informed consent prior to their participation. The study protocol was approved by the University of Pittsburgh Institutional Review Board, was reviewed annually, and was registered in the national clinical trials database. There were 31 participants included in the larger randomized feasibility trial of CET (18), with 14 subjects (*n* = 10 CET and *n* = 4 TAU) available for functional magnetic resonance imaging (fMRI) after completing treatment. The reasons for participants being unavailable for scanning included withdrawing consent (*n* = 5), incarceration (*n* = 1), cocaine abuse (*n* = 1), symptom instability (*n* = 4), heroin dependence (*n* = 1), lack of interest in being scanned (*n* = 2), unable to contact (*n* = 2), and ferromagnetic objects in the body (*n* = 1). Inclusion criteria for the participants were (1) age between 18 and 60, (2) diagnosis of schizophrenia or schizoaffective disorder based on the Structured Clinical Interview for the DSM-IV [SCID (30)], (3) presentation of significant cognitive and social disability on the Cognitive Styles and Social Cognition Eligibility Interview (31), (4) an Addiction Severity Index (32) score of moderate or higher (≥4) addiction severity for cannabis or alcohol use, (5) stabilization on antipsychotic medications, (6) an IQ ≥ 80, (7) the ability to speak and read fluent English, (8) no current cocaine, amphetamine, or opioid abuse or dependence, (9) not receiving substance abuse pharmacotherapies (e.g., naltrexone), (10) no significant cognitive impairment caused by the presence of a persistent medical condition, (11) no persistent suicidal or homicidal behavior, and (12) free of any MRI contraindications.

See Table 1 for a full description of participant characteristics. Overall, participants with fMRI data had an average age of 38.71 (SD = 13.26) years, were ill for an average duration of 14.93 (SD = 10.38) years, and completed 14.50 (SD = 1.61) years of education. Eighty-six percent (*n* = 12) of the participants also had a comorbid alcohol and/or cannabis misuse diagnosis based on the SCID (30). Two participants did not meet SCID criteria for a comorbid alcohol and/or cannabis misuse diagnosis, but met for the inclusion criteria of an Addiction Severity Index (32) score of moderate or higher (≥4) addiction severity for cannabis or alcohol use. The participants were ethnically diverse (seven Caucasian, six African American, and one Asian) and a little more than half were male (*n* = 8, 57%). Although the majority of the participants had some college education (*n* = 11, 79%) and most were not employed (*n* = 11, 79%) at the time of baseline assessment. Participants randomized to either CET (*n* = 10) or TAU (*n* = 4) did not significantly differ with regard to the above demographic variables (all *p* > 0.271), baseline IQ (*p* = 0.541), BPRS total score (*p* = 0.258), antipsychotic medication dose (*p* = 0.464), type of antipsychotic mediation (0.505), or adherence to antipsychotic medication (*p* = 1.00). The observation of a non-significant difference between CET and TAU participants with regard to type of antipsychotic medication is important given that typical and atypical antipsychotics may impact brain functioning differently (33). The CET participants were non-significantly older than the TAU participants, and there were more Caucasian participants in the CET group (*n* = 6, 60%) compared to only one (25%) Caucasian participant in the TAU group. Consequently, age and race were included as confounding covariates in all analyses of the differential effects of CET compared to TAU on brain function.

**Table 1:** **Baseline characteristics of CET and TAU participants with schizophrenia who misuse alcohol and/or cannabis presented as *N* (%) or *M* (SD)**.

Characteristic	Total (*n* **=** 14)	CET (*n* **=** 10)	TAU (*n* **=** 4)	*p*^a^
Age (years)	38.7 (13.26)	41.20 (13.65)	32.50 (11.45)	0.285
Sex (male)	8 (57%)	6 (60%)	2 (50%)	1.00
Race: Caucasian	7 (50%)	6 (60%)	1 (25%)
African-American	6 (43%)	4 (40%)	2 (50%)	0.271
Asian	1 (7%)	–	1 (25%)
IQ	101.43 (11.69)	102.70 (13.6)	98.25 (4.27)	0.541
Attended college	11 (79%)	8 (80%)	3 (75%)	0.728
Education (years)	14.50 (1.61)	14.6 (1.65)	14.25 (1.71)	1.00
Not employed	11 (79%)	7 (70%)	4 (100%)	0.505
Illness length (years)	14.93 (10.38)	15.50 (10.52)	13.5 (11.45)	0.759
BPRS total	42.93 (10.26)	40.90 (7.77)	48.0 (15.08)	0.258
ASI: alcohol	4.5 (2.68)	4.40 (3.10)	4.75 (1.50)	0.835
ASI: drug	3.14 (2.14)	3.0 (2.49)	3.50 (1.00)	0.710
Principle diagnosis				
Schizophrenia	10 (71%)	8 (80%)	2 (50%)
Schizoaffective	4 (29%)	2 (20%)	2 (50%)	0.520
Substance abuse or dependence diagnosis	12 (86%)	9 (90%)	3 (75%)	0.505
Alcohol dependence	7 (50%)	5 (50%)	2 (50%)	1.00
Alcohol abuse	2 (14%)	2 (20%)	–	1.00
Cannabis dependence	7 (50%)	5 (50%)	2 (50%)	1.00
Cannabis abuse	1 (7%)	–	1 (25%)	0.286
Daily substance use among active users				
Alcohol use occasions per day	2.15 (2.39)	2.59 (2.42)	1.07 (2.14)	0.301
Cannabis use occasions per day	0.34 (0.85)	0.48 (0.98)	–	0.363
Antipsychotic medication				
Atypical	11 (79%)	7 (70%)	4 (100%)	0.505
Typical	3 (21%)	3 (30%)	–	
Dose (CPZ equivalent)	449.52 (360.69)	402.67 (370.68)	566.67 (354.86)	0.464
Adherent	13 (93%)	9 (90%)	4 (100%)	1.00
MSCEIT total score				
Baseline	87.33 (14.98)	85.94 (14.27)	90.82 (18.40)	0.602
Posttreatment	91.17 (15.53)	92.19 (14.98)	88.61 (18.96)	0.713
ER-40 correct response				
Baseline	32.29 (3.45)	31.90 (3.96)	33.25 (1.71)	0.530
Posttreatment	33.18 (3.05)	33.35 (3.54)	32.75 (1.50)	0.754

*ASI, Addiction Severity Index; BPRS, Brief Psychiatric Rating Scale; CPZ, chlorpromazine, MSCEIT, The Mayer–Salovey–Caruso Emotional Intelligence Test; ER-40, Penn Emotion Recognition Test-40*.

*^a^Results from independent sample *t*-tests or Fisher’s exact tests, two-tailed*.

### Emotional Faces *n*-Back Task

**Figure 1:**
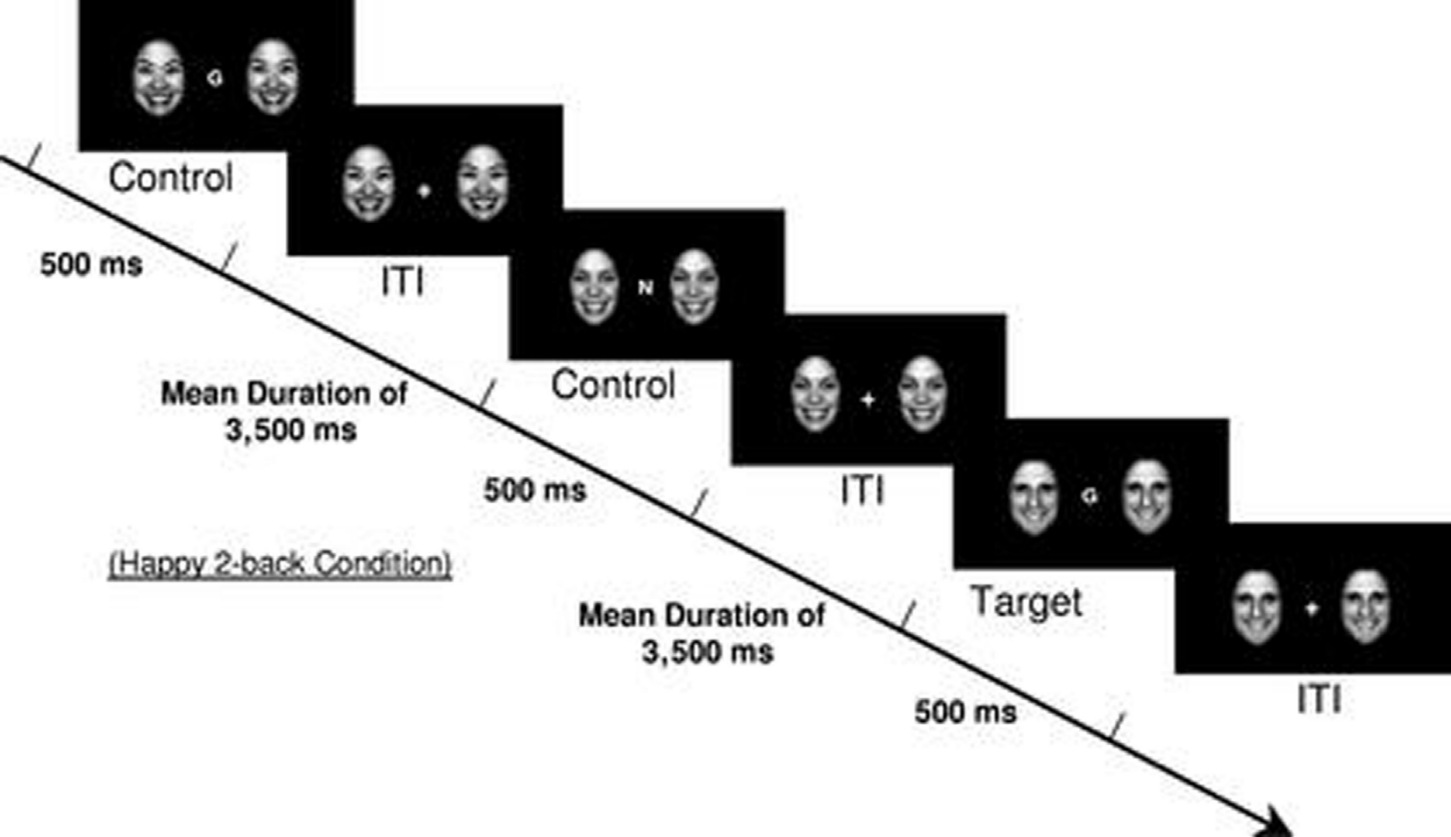
**Example of a 2-back happy face distracter condition from the emotional faces *n*-back task**. [Reproduced with permission from Ladouceur et al. (34)].

Brain functioning during the effortful regulation of emotion was elicited using an emotional faces *n*-back task, which is a modified version of the standard working memory *n*-back task including 0-back and 2-back working memory conditions (34). An *n*-back task is one that asks participants to respond when they view a stimulus (e.g., letter) that is the same as that presented *n* trails previously. In addition to the working memory conditions (0-back and 2-back), four emotional valence distracter conditions (no faces, happy faces, fearful faces, and neutral faces) were presented to participants. Faces were flanked on each side of the *n*-back letter stimuli (Figure 1). The faces were from the NimStim dataset in grayscale (35), were normalized for size and luminance, and balanced by gender. Both working memory and emotional valence conditions were presented in randomized blocks of 12 trials each. By directing participants’ attention to the working memory components of the task, participants were required to inhibit their response to the emotional stimuli in order to successfully complete the *n*-back tasks (36), and thus this task is considered a test of effortful emotion regulation. Each block started with instructions indicating the working memory condition (0-back or 2-back) presented on the screen for 3500 ms, which was followed by the target stimulus (letters) presented flanked by the different emotional valence distracter conditions (happy, fearful, neutral, or no face) for 500 ms, with an interstimulus interval jittered at an average of 3500 ms. The total task time was 6 min and 56 s with each emotional valence distracter condition presented once for both the 0-back and 2-back conditions. All the participants completed two runs of the emotional faces *n*-back paradigm, with the exception of one TAU participant that completed one run. Since the focus of this research was on emotion regulation during effortful cognitive processing, only data from the effortful 2-back condition were analyzed.

### Image Acquisition and Processing

A 3-T Siemens Verio whole-body scanner with a 12-channel head coil was used to collect structural and functional neuroimaging data at the Scientific Imaging and Brain Research Center at Carnegie Mellon University. Functional MRI data were acquired using an echo T2*-weighted sequence with real-time motion correction (3.2 mm × 3.2 mm × 3.2 mm voxel size, TR = 2000 ms, TE = 30 ms, bandwidth = 2298 Hz/px, FOV = 205 mm, flip angle = 79°, 64 × 64 matrix, 36 slices, slice thickness = 3.2 mm).

Statistical Parametric Mapping Software, version 8 (SPM8; Wellcome Department of Cognitive Neurology, Institute of Neurology, London, UK) was used to preprocess imaging data. The images were normalized to a common Montreal Neurological Institute (MNI) coordinate space using an indirect normalization pipeline, with parameters obtained from a high-resolution T1 structural image. Images were smoothed with an 8-mm full-width at half maximum Gaussian kernel. Signal and movement outliers were identified using the Artifact Detection Tool (37) software package and entered into each participant’s first-level general linear model as regressors of no interest.

### Emotion Processing Behavioral Measures

In addition to assessing brain functioning during effortful emotion regulation measured by the emotional faces *n*-back task outlined above, behavioral measures of emotion processing were also utilized in the parent study (18) to examine behavioral emotion processing skills at pre- and posttreatment. The Mayer–Salovey–Caruso Emotional Intelligence Test [MSCEIT (38)] and the Penn Emotion Recognition Test-40 [ER-40 (39)] were the measures used to assess emotion processing. Noteworthy, brain regions important for emotion processing, which includes emotion regulation, such as limbic areas, have been shown to be associated with performance on both these measures (40, 41).

The MSCEIT is a 141-item performance-based measure of emotion processing and management (e.g., emotional intelligence) that is administered on the computer (42). Performance scores are based on a large normative sample scale (*M* = 100, SD = 15), with a higher score indicating a better emotional intelligence quotient (38). The MSCEIT is a recommended measure of the National Institute of Mental Health’s committee on Measurement and Treatment Research to Improve Cognition in Schizophrenia [NIMH-MATRICS (43)] and has been shown to have good reliability and sufficient construct and concurrent validity (43, 44). The ER-40 is also a frequently utilized measure of emotion processing in schizophrenia, as it has good psychometric properties (45) and is able to discriminate between healthy controls and individuals with schizophrenia (39). The ER-40 is a forced-choice, computer-administered assessment of the ability to accurately recognize different emotions. The task involves a series of 40 happy, sad, angry, fearful, or neutral (non-emotional) faces. Scores represent number of correct responses, with a higher score indicating better performance.

## Treatments

### Medication

All participants were maintained on antipsychotic medications approved for the treatment of schizophrenia or schizoaffective disorder as prescribed by their treating psychiatrist. Table 1 lists antipsychotic medication characteristics of the participants.

### Cognitive Enhancement Therapy

Cognitive enhancement therapy is a performance-based, comprehensive, developmental approach to the remediation of social-cognitive and neurocognitive deficits in participants with schizophrenia (17). CET consists of 60 h of weekly computer-based neurocognitive training to in attention, memory and problem-solving and 45 small-group sessions to address social-cognitive deficits that limit functional recovery from schizophrenia. To encourage socialization, neurocognitive training is implemented in patient pairs and conducted with coaching from a CET therapist. One-hour neurocognitive training sessions begin with Ben-Yishay’s Orientation Remediation Module (46) to improve different aspects of attention and speed of processing. Following ~3 months of attention training, 3–4 participants combine to form a social-cognitive group. The 1.5-h social-cognitive group sessions utilize experiential learning approaches to teach a wide range of social-cognitive abilities designed to enhance social wisdom and interpersonal success. Key theoretically driven components of the social-cognitive groups include perspective-taking, social gist abstraction, non-verbal communication, emotion management, and foresightfulness. The social-cognitive group curriculum encourages participants to engage in activities that include responding to unrehearsed social exchanges, presenting homework, participating in social-cognitive exercises, providing feedback to others, and leading homework review. Neurocognitive training in memory and problem-solving using PSSCogReHab (47) software proceeds concurrently with the social-cognitive groups after the conclusion of attention training. A complete description of CET has been provided elsewhere (17).

### Treatment As Usual

Treatment as usual served as the comparison treatment condition in this randomized feasibility trial of CET for schizophrenia participants who misuse substances. TAU consisted of traditional social services and mental health programs, which included psychiatric services, case management, individual supportive therapy, vocational rehabilitation, dual diagnosis treatment programs, and other community-based treatments for substance use. All efforts were made to link both CET and TAU participants with necessary mental health and substance abuse services while participating in the study.

## Procedures

Participants were recruited from Western Psychiatric Institute and Clinic and other community clinics at Pittsburgh, PA, USA. Potential participants were screened for eligibility using the SCID (30), the Ammons Quick IQ Test (48), the Addiction Severity Index (32), and the Cognitive Style and Social Cognition Eligibility Interview (31). Those participants meeting inclusion criteria were then randomized to receive 18 months of either CET or TAU. Individuals were then assessed every 6 months using the aforementioned behavioral emotion processing measures. A subset of 14 participants (*n* = 10 CET, *n* = 4 TAU) completed posttreatment fMRI scanning. Neuroimaging assessments were only available posttreatment as part of a separate pilot study that became available near the end of the larger feasibility trial of CET for alcohol and/or cannabis misusing schizophrenia (18). See Eack et al. (18) for details on this larger feasibility trial, such as the randomization procedures and the enrollment diagram. All participants provided written informed consent prior to their participation. The study protocol was approved by the University of Pittsburgh Institutional Review Board, was reviewed annually, and was registered in the national clinical trials database (NCT01292577).

### Data Analysis

Functional neuroimaging data are inherently hierarchical in nature, with brain images (collected every 2 s) nested within individuals in a time series. Analysis proceeds by first estimating the effects of task condition on brain activity for each individual (first-level analysis) and then subjecting those contrasts to group (second-level) analyses (49). SPM8 was utilized for first- and second-level voxel-based analyses to examine the differential posttreatment effects of CET compared to TAU on emotion regulation-related brain functioning. First-level analyses consisted of modeling neural responses during each condition of the emotional faces *n*-back task with general linear models in each of the participants. First-level models also included the signal and motion outliers identified by the Artifact Detection Tool as covariates (37). First-level contrasts (happy face vs. no face, fearful face vs. no face, and neutral face vs. no face) were then entered into a second-level group analysis based on a two (CET vs. TAU) × three (happy face vs. no face, fearful face vs. no face, and neutral face vs. no face) general linear model. To control the effect of general visual stimulation, the second-level contrasts compared emotional faces to neutral faces [e.g., (happy vs. no face)–(neutral vs. no face)]. As mentioned above, second-level models included age and race as confounding covariates. A single region of interest mask was created in the Wake Forest University PickAtlas toolbox (50) with anatomical definitions provided by Tzourio-Mazoyer, Landeau (51). Regions of interest included frontolimbic and striatal areas, which were the bilateral amygdala, insula, dorsolateral prefrontal cortex (DLPFC), ventromedial prefrontal cortex, orbitofrontal cortex, striatum, nucleus accumbens, and the anterior cingulate cortex. These regions have been repeatedly implicated in the regulation of emotion (22, 24, 36, 52). Due to the conservativeness of voxel-wise multiple comparison corrections in small samples (53), Type I error was controlled using a cluster-extent thresholding method. Cluster-level correction with a small sample may provide the best balance between type I and type II error (54). Based on 10,000 Monte Carlo simulations executed in 3dClustSim (55), type I error was indicated to be controlled at an α-level of 0.05 using a combined threshold of a voxel extent of 34 and an uncorrected *p* of 0.001.

Associations between differential posttreatment effects during emotion regulation-related brain functioning and changes in behavioral emotion regulation performance from pre- to posttreatment were analyzed with bivariate correlations and mediator models executed in R 3.1.2 (56). It was determined that age, race, and antipsychotic medication dose were not significantly related to emotion processing behavioral performance and emotion regulation-related brain functioning. Therefore, these variables were not included as covariates to retain statistical power in correlations and mediator models. Average magnitude estimates per region of interest per participant were extracted using MarsBar, version 0.44 [http://marsbar.sourceforge.net (57)] from the above imaging analysis to be utilized for correlation and mediation analyses. A path analysis approach was used for the mediation analysis (58), which was based on the mediator-analytic framework presented by Kraemer et al. (59) for randomized clinical trials. Mediator models were constructed with a series of linear models (60) that analyzed the indirect effects of treatment assignment (predictor) on longitudinal changes in emotion processing behavioral performance (outcome) through posttreatment emotion regulation-related brain functioning (mediator). This was accomplished by computing the association between (1) treatment assignment and emotion regulation-related brain functioning, (2) emotion regulation-related brain function and longitudinal changes in emotion processing behavioral performance, and (3) treatment assignment and longitudinal changes in emotion processing behavioral performance. Based on MacKinnon et al. (61), the magnitude and significance of the mediation effects was estimated using an asymptotic *z*′ test of indirect effects. Logarithmic transformations were used to correct any variables with significantly skewed distributions prior to analysis, which included the left inferior orbital frontal cortex, right DLPFC (BA 46), and reaction time from the emotional faces *n*-back. Missing data were handled with an expectation-maximization approach (62).

## Results

### Emotional Faces *n*-Back Task Performance

Reaction time and accuracy performance on the emotional faces *n*-back task was also analyzed in R with mixed-effects models examining group (CET vs. TAU), working memory loading (0-back vs. 2-back) and emotional distracter valence (happy vs. fearful vs. neutral vs. no faces) effects. One TAU participant had accuracy data available but did not have recorded reaction time data due to technical issues. Investigation of task performance on the emotional faces *n*-back task during scanning revealed that participants had neither overall high accuracy, with no significant group (*p* = 0.228) or emotional distracter valence differences (*p* = 0.803) nor were there any significant group by emotional distracter valence interactions (*p* = 0.921), group by working memory loading interactions (*p* = 0.369), or group by emotional distracter valence by working memory loading interactions (*p* = 0.918). Accuracy was high across both groups of participants, however, significantly lower for the 2-back condition (93%) compared to the 0-back condition (97%), **χ**^2^ (1, *N* = 14) = 9.68, *p* = 0.002.

Overall, participants did not significantly differ in reaction times with regard to group assignment (*p* = 0.695). With regard to the working memory loading condition, the participants had significantly slower reaction times during the 2-back condition (*M* = 6.73 ms_log_, SE = 0.06) compared to the 0-back condition (*M* = 6.44 ms_log_, SE = 0.06), **χ**^2^ (1, *N* = 13) = 72.81, *p* < 0.001. CET and TAU participants had similar reaction times during the 0-back (CET: *M* = 6.44 ms_log_, SE = 0.05; TAU: *M* = 6.42 ms_log_, SE = 0.10), but during the 2-back condition, the CET participants had significantly slower reaction times (*M* = 6.78 ms_log_, SE = 0.05) compared to the TAU participants (*M* = 6.68 ms_log_, SE = 0.10), **χ**^2^ (1, *N* = 13) = 3.86, *p* = 0.049. Participants did neither significantly differ in reaction times with regard to emotional valence distracters (*p* = 0.058) nor were significant interactions observed for group by emotional distracter valence (*p* = 0.415) or group by emotional distracter valence by working memory loading (*p* = 0.491). Such results are confirmatory that participants were paying attention to the task and any differences in brain functioning elicited during the task are not due to differential inability to complete the task.

### Posttreatment Effects of CET on Frontolimbic and Striatal Brain Functioning During Effortful Emotion Regulation

**Figure 2:**
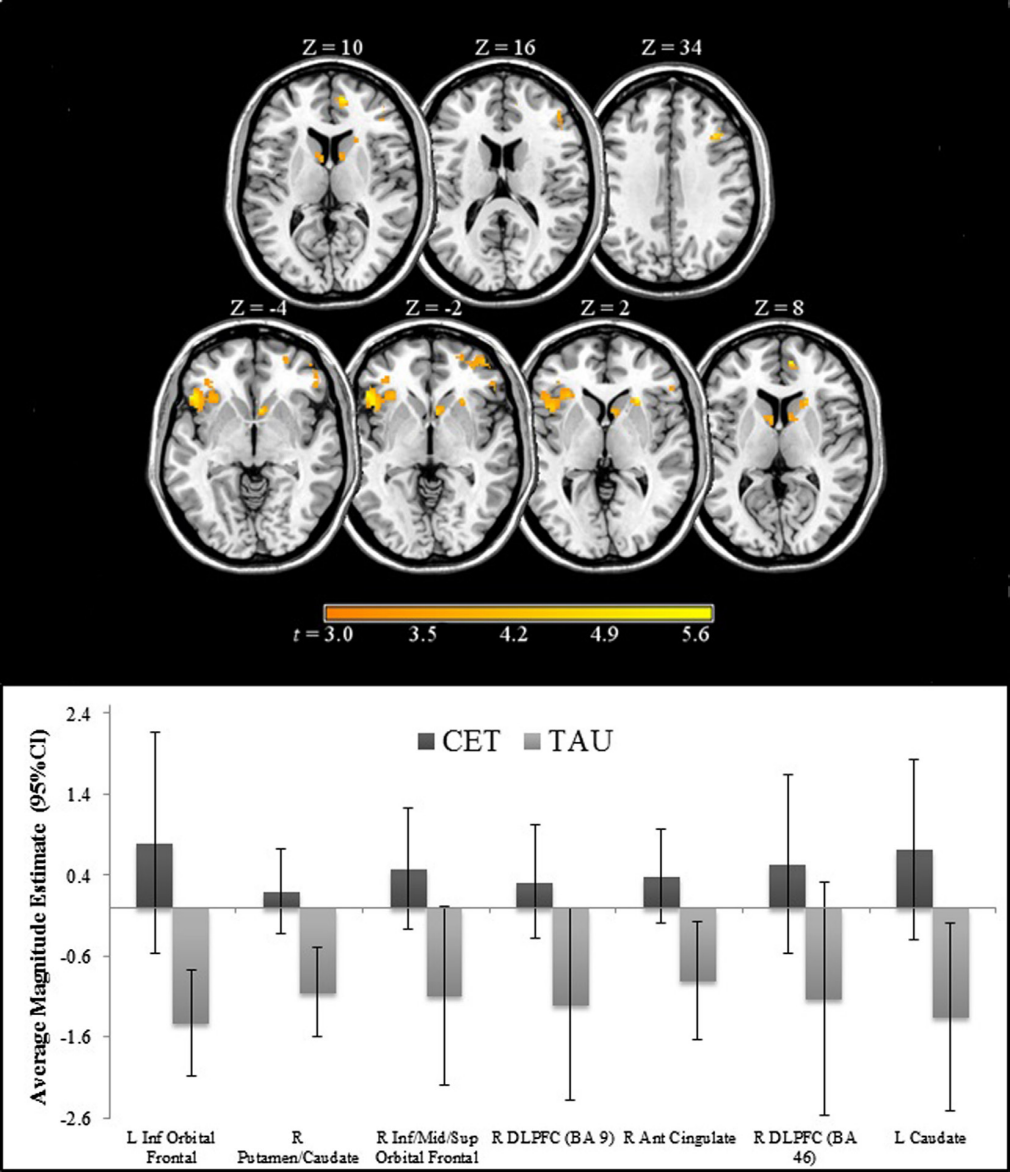
**Regions of significantly greater activation in participants completing 18 months of CET, compared to TAU, during the emotional faces *n*-back task**.

Region-of-interest voxel-based analyses were conducted using a 2 (CET vs. TAU) × 3 (happy vs. no face, fearful vs. no face, and neutral vs. no face) general linear model to investigate posttreatment brain differences between CET and TAU participants during effortful emotion regulation. No significant interaction effects were observed with regard to the emotional distracter conditions, and thus the main effects of treatment group were examined. Compared to the TAU group, CET participants displayed significantly greater activation during the emotion regulation task in a large cluster involving the left inferior orbital frontal, insula, and ventromedial prefrontal cortices (Table 2; Figure 2). Participants treated with CET, compared to TAU, also had significantly greater emotion regulation-related activation in the right DLPFC, right anterior cingulate cortex, right putamen, bilateral caudate, and in a moderately sized cluster in the right orbital frontal and right ventromedial prefrontal cortices (Table 2; Figure 2). Accordingly, the direction of greater activation related to CET during emotion regulation in the above frontolimbic and striatal regions may be indicative that CET is contributing to neurobiological changes in people with schizophrenia and comorbid substance misuse problems.

**Table 2:** **Differential activation during the emotional faces *n*-back task observed between CET and TAU participants at posttreatment**.

Region	BA	MNI coordinates	Cluster size	*z*	*p*	Direction
L inferior orbital frontal	47, 13	−50 24 −4	530	4.61	<0.001	CET > TAU
L insula						
L ventromedial prefrontal						
R putamen/caudate	–	26 22 2	160	4.56	<0.001	CET > TAU
R inferior/middle/superior orbital frontal	47	44 52 −2	91	3.73	<0.001	CET > TAU
R ventromedial prefrontal						
R DLPFC	9	38 24 34	69	4.13	<0.001	CET > TAU
R anterior cingulate	10	12 52 10	43	4.67	<0.001	CET > TAU
R DLPFC	46	46 40 16	38	3.49	<0.001	CET > TAU
L caudate	–	−6 6 8	37	3.55	<0.001	CET > TAU

*BA, Brodmann area; MNI, Montreal Neurological Institute; L, left; R, right; CET, Cognitive Enhancement Therapy; TAU, treatment as usual*.

*Presented results are corrected for multiple comparisons based on 10,000 Monte Carlo simulations executed in AlphaSim (55) using 3dClustSim (α-level of 0.05, *k* = 34, uncorrected *p* of 0.001)*.

### Association Between Posttreatment Brain Functioning During Emotion Regulation and Longitudinal Improvements in Emotion Processing Behavioral Performance

Greater posttreatment activation during the effortful regulation of emotion in all regions observed to be significantly different between CET and TAU participants (Table 2) were significantly correlated with greater longitudinal improvements in MSCEIT total scores (all *r*’s 0.58–0.77, all *p* < 0.030), with the exception of the right DLPFC (BA 46 only). Greater longitudinal improvement on the ER-40 was significantly correlated with greater posttreatment activation in the large cluster involving the left inferior orbital frontal, ventromedial prefrontal, and insula cortices (*r* = 0.56, *p* = 0.037). Greater activation in the orbital frontal cortex (cluster including the inferior, middle, and superior levels; *r* = 0.65, *p* = 0.011) and the right DLPFC (BA 9; *r* = 0.56, *p* = 0.038) was also significantly associated with greater longitudinal improvement on the ER-40. Mediation analyses revealed that, when adjusting for treatment assignment, greater differential emotion regulation-related activation, favoring CET, in the cluster involving the left inferior orbital frontal, ventromedial prefrontal, and insula cortices, had a significant direct effect on improved total scores on the MSCEIT, with this cluster significantly mediating the association between treatment assignment and improved performance on this test (Table 3). No direct or mediation effects were observed with regard to changes scores on the ER-40.

**Table 3:** **Relationships between posttreatment emotion regulation brain functioning and longitudinal changes in behavioral emotion processing performance**.

	Direct effect					Mediator effect
	
Regional clusters	***B***	SE	***t***	df	***p***	*z***′**
	ΔMSCEIT total score					
L inferior orbital frontal
L insula	14.3	4.3	3.3	11	0.007	−2.7**
L ventromedial prefrontal
R putamen/caudate	5.6	3.0	1.9	11	0.086	−1.7^+^
R inferior/middle/superior orbital frontal	3.1	2.1	1.5	11	0.169	−1.2
R ventromedial prefrontal						
R DLPFC (BA 9)	3.7	2.2	1.7	11	0.116	−1.4
R anterior cingulate	4.3	2.8	1.5	11	0.155	−1.4
R DLPFC (BA 46)	1.4	5.9	0.2	11	0.813	−0.2
L caudate	2.8	1.4	2.1	11	0.062	−1.8^+^
	ΔER-40 correct responses					
L inferior orbital frontal
L insula	1.6	1.3	1.2	11	0.251	−1.2
L ventromedial prefrontal
R putamen/caudate	0.4	0.8	0.6	11	0.578	−0.6
R inferior/middle/superior orbital frontal	0.9	0.5	2.0	11	0.072	−1.6
R ventromedial prefrontal						
R DLPFC (BA 9)	0.7	0.5	1.3	11	0.206	−1.1
R anterior cingulate	0.2	0.7	0.3	11	0.764	−0.3
R DLPFC (BA 46)	1.4	1.3	1.1	11	0.292	−0.9
L caudate	0.3	0.4	0.8	11	0.443	−0.8

*L, left; R, right; DLPFC, dorsal lateral prefrontal cortex; MSCEIT, The Mayer–Salovey–Caruso Emotional Intelligence Test; ER-40, Penn Emotion Recognition Test-40*.

**p < 0.01.

*^+^*p* < 1.00*.

## Discussion

Substance misuse among people with schizophrenia, especially for alcohol and cannabis (2), is a common, significant problem as addiction is associated with more severe illness trajectories (11) and worse community functioning (6). Poor emotion regulation may be a key contributor of elevating the risk for substance misuse in individuals with schizophrenia (3, 4). Neural correlates of disrupted emotion regulation in individuals with schizophrenia and substance misuse problems (52) have been shown to include frontal, limbic, and striatal regions important for emotional neurocircuitry (22, 23, 36). Recently, significant improvements in emotion regulation abilities were observed in individuals with schizophrenia who also misuse alcohol and/or cannabis after being treated with CET, a cognitive remediation intervention (18). Therefore, this original, exploratory study sought to examine differences in brain functioning during effortful emotion regulation in participants with comorbid schizophrenia and alcohol and/or cannabis misuse following CET or TAU. The direct and mediation effects of these neurobiological differences on longitudinal changes in behavioral emotion processing outcomes were also examined.

Compared to participants in TAU, CET participants displayed significantly greater activation in frontal, limbic, and striatal networks involved in the regulation of emotion at posttreatment (22–24, 36, 63), including the DLPFC, ventromedial prefrontal cortex, orbital frontal cortex, anterior cingulate, insula, caudate, and putamen. No significant interactions were observed regarding emotional valence during the emotional faces *n*-back task. Longitudinal improvements in behavioral emotion processing abilities were correlated with greater activation in the majority of these above regions. Interestingly, a mediating effect was observed in an area including the orbital frontal cortex, ventromedial prefrontal cortex, and the insula such that greater brain activation in these regions mediated longitudinal improvements in behavioral emotion processing abilities.

Such results suggest that treatment with CET (18) may be normalizing the coordination and function of frontolimbic and striatal regions involved in emotion regulation in individuals with schizophrenia who also misuse alcohol and/or cannabis. This is evidenced by research demonstrating that communication of prefrontal and limbic regions modulates cognitive control over emotion regulation abilities (64–66), which has been observed to be dysregulated in individuals with schizophrenia (67, 68). It may be that improved cognitive functioning gained through CET (69, 70) increased participants’ ability to regulate and manage their emotional states. Meta-analytic evidence has shown that cognitive remediation interventions have a common neural plasticity effect of increasing activation in frontal and limbic regions that are related to improved cognitive and socio-emotional functioning in individuals with schizophrenia (27). The findings from this investigation of increased task-related activation in some overlapping frontolimbic regions are supportive of CET as an effective intervention for supporting functional recovery of this underserved, vulnerable population.

Of course, these findings have many caveats that preclude firm conclusions regarding causality of treatment efficacy. The first limitation is the very small sample size employed in this research, particularly in the TAU condition. This may explain the lack of significant emotional valence interactions with group assignment from the emotional faces *n*-back task, although sufficient power was available to detect the very large effects observed in frontolimbic brain functioning. Also related to the small sample size, the groups were not perfectly matched especially with regard to age and race. Although age and race were not significantly different between the groups, we did include them as possible confounders in all analyses examining differential effects of treatment on brain functioning. Noteworthy, CET had a higher, but non-significantly different attrition rate compared to TAU in the larger feasibility trial [for a further description, see Eack et al. (18)]. Next, because the imaging component was an opportunistic add-on study funded near the completion of the parent clinical trial, no pretreatment imaging data were available. Posttreatment randomized-controlled trials are common and protect against many threats to internal validity (71), but in the case of quantitative outcomes with unknown baseline values, they are unable to determine the magnitude of change. We suspect that the greater brain activation observed in CET during emotion regulation is reflective of longitudinal increases in frontolimbic activity. However, in the absence of an active comparison group, such findings may also reflect non-specific CET effects associated with more therapeutic contacts, including group activities. Further, retention of participants for fMRI procedures was greater in CET than TAU, which may have impacted the results by limiting the sample of TAU participants. However, there were no significant differences in baseline characteristics observed between TAU participants who did vs. did not complete an fMRI scan (*p* > 0.166). In addition, we did not assess participants level of alcohol and/or cannabis use at the time of posttreatment scanning or during assessments of emotion processing, which could have influenced brain functioning and performance. It will be important for future studies to employ longitudinal imaging methods and assess substance misuse at the time of assessments to address these issues, and until these findings can be replicated in adequately powered samples they should be considered tentative and interpreted with caution. It will also be important for future research with larger sample sizes to examine the implications of such findings on symptom severity and other functional outcomes in the illness.

In summary, this preliminary study was the first to show a possible neural plasticity relationship between CET and emotion regulation-related brain functioning in individuals with schizophrenia and alcohol and/or cannabis misuse comorbidities participating in a randomized clinical trial. The findings indicated that CET may lead to differential changes in functioning of frontolimbic and striatal regions implicated in the regulation of emotion. Increased activation in these regions during effortful emotion regulation was supportive of longitudinal improvements in behavioral emotion processing abilities. Improved emotion regulation may serve as a protective factor for substance misuse as well as play a role in improved interpersonal and other psychosocial functioning. The findings from this investigation are not only informative for future research but also highlight the utility of providing cognitive remediation interventions, such as CET, to optimize recovery for people with schizophrenia who have substance misuse problems, particularly for alcohol and cannabis.

## Author Contributions

JW, SH, JC, MP, MK, CN, and SE all made substantial contribution to this manuscript.

## Conflict of Interest Statement

The authors declare that the research was conducted in the absence of any commercial or financial relationships that could be construed as a potential conflict of interest.
